# Regional and Temporal Differences in Brain Activity With Morally Good or Bad Judgments in Men: A Magnetoencephalography Study

**DOI:** 10.3389/fnins.2021.596711

**Published:** 2021-04-12

**Authors:** Hirotoshi Hiraishi, Takashi Ikeda, Daisuke N. Saito, Chiaki Hasegawa, Sachiko Kitagawa, Tetsuya Takahashi, Mitsuru Kikuchi, Yasuomi Ouchi

**Affiliations:** ^1^Department of Biofunctional Imaging, Preeminent Medical Photonics Education and Research Center, Hamamatsu University School of Medicine, Hamamatsu, Japan; ^2^Research Center for Child Mental Development, Kanazawa University, Kanazawa, Japan; ^3^United Graduate School of Child Development, Osaka University, Kanazawa University, Hamamatsu University School of Medicine, Chiba University and University of Fukui, Osaka, Japan; ^4^Department of Psychology, Yasuda Women’s University, Hiroshima, Japan; ^5^Department of Psychiatry and Behavioral Science, Kanazawa University, Kanazawa, Japan

**Keywords:** moral judgment, MEG, brain activity, connectivity, morally good judgment, morally bad judgment

## Abstract

Many neuroimaging studies on morality focus on functional brain areas that relate to moral judgment specifically in morally negative situations. To date, there have been few studies on differences in brain activity under conditions of being morally good and bad along a continuum. To explore not only the brain regions involved but also their functional connections during moral judgments, we used magnetoencephalography (MEG), which is superior to other imaging modalities for analyzing time-dependent brain activities; only men were recruited because sex differences might be a confounding factor. While analyses showed that general patterns of brain activation and connectivity were similar between morally good judgments (MGJs) and morally bad judgments (MBJs), activation in brain areas that subserve emotion and “theory of mind” on the right hemisphere was larger in MGJ than MBJ conditions. In the left local temporal region, the connectivity between brain areas related to emotion and reward/punishment was stronger in MBJ than MGJ conditions. The time-frequency analysis showed distinct laterality (left hemisphere dominant) occurring during early moral information processing in MBJ conditions compared to MGJ conditions and phase-dependent differences in the appearance of theta waves between MBJ and MGJ conditions. During MBJs, connections within the hemispheric regions were more robust than those between hemispheric regions. These results suggested that the local temporal region on the left hemisphere is more important in the execution of MBJs during early moral valence processing than in that with MGJs. Shorter neuronal connections within the hemisphere may allow to make MBJs punctual.

## Introduction

Moral judgment can be defined as the evaluation of actions with respect to norms and values established in a society (such as not stealing or being an honest citizen), and when judging a behavior as morally good or bad, people refer to their internal representations of these norms and values (i.e., emotionally laden internal moral orientations or principles) (Prehn et al., 2008). While we judge our behaviors as being morally good or bad frequently in our daily lives, the primary concern in many previous neuroimaging studies was related to conditions of being morally bad, and the stimuli were visually presented with sentences and/or pictures that participants were asked to judge with regard to their moral valence (Farrow et al., 2001; Greene et al., 2001; Moll et al., 2002a,b; Heekeren et al., 2005; Takahashi et al., 2008). Indeed, neuroimaging studies on morality often used negative stories with/without a moral context that contained different features (i.e., trolley dilemma vs footbridge dilemma). To the best of our knowledge, there are several neuroimaging studies examining both valences: morally positive (prosocial/helping) and morally negative (antisocial/harming) (Takahashi et al., 2008; Hayashi et al., 2014; Cowell and Decety, 2015). Largely, tasks used in these kinds of studies are categorized into two types: morally thinking or judging about what to do in a hypothetical moral dilemma (i.e., trolly dilemma task) and morally evaluating or judging whether the situation is permissible or unacceptable (Garrigan et al., 2016). To simulate situations similar to our daily lives, the latter type of moral study was considered suitable to our present study design where time-dependent changes in brain activity while making judgments of a situation being morally good or bad.

Brain regions that relate to morality are the prefrontal, precuneus, superior temporal and anterior cingulate cortices, insula, thalamus and amygdala, many of which are considered important in the regulation of sociality, emotion, empathy, “theory of mind,” decision-making and reward/punishment (Greene and Haidt, 2002; Moll et al., 2003). Judgment of situations being morally unacceptable occurred predominantly in the left hemisphere (Cope et al., 2010), and intent-based moral judgment needed the recruitment of the right hemisphere in an investigation of patients with a split-brain (Steckler et al., 2017). Currently, the role of hemispheric laterality in good or bad moral decisions remains unclear.

It is not only particular brain regions but also neural connectivity that is important in the exploration of moral judgment in a time-dependent manner. Using electroencephalography (EEG), characteristic wave forms such as N400 in Cz-Pz (Kutas and Hillyard, 1980) and late positive component (Hajcak and Nieuwenhuis, 2006) are developed during moral judgments. An EEG study by Decety and Cacioppo (2012) showed that moral information might proceed from the posterior-superior temporal sulcus (62–140 ms) through the amygdala (122–180 ms) and to the ventromedial prefrontal cortex (182–304 ms), mainly in the right hemisphere. The merit of EEG is this capability to measure the timing of changes in brain activation. Likewise, magnetoencephalography (MEG) is a noninvasive neuroimaging method that allows the measurement of ongoing brain activity at the same time resolution as EEG (millisecond order) and is superior to EEG in acquiring more detailed information about particular brain regions. Functional connectivity between brain regions has been defined as the temporal correlation between spatially remote neurophysiological events (Friston et al., 1993). Brain imaging paradigms assessing functional connectivity can easily be conducted using high time-resolution techniques such as EEG and MEG on the basis that if the activation of two brain regions in response to a task is correlated, then they are functionally connected (Menon et al., 2001; Meyer-Lindenberg et al., 2001; Lawrie et al., 2002). This technique is indeed advantageous in a functional study with a paradigm consisting of moral tasks.

In the present study, we aimed to explore functional brain activity and connectivity in the specific regions during morally good judgments (MGJs) and morally bad judgments (MBJs) using MEG that allows to examine both regional cerebral activation and time-dependent changes. We hypothesized that brain processes in these two judgments are different in the cerebral hemisphere-dependent and time-dependent manners, specifically in the temporal cortex because of the current tasks we used about morality with the verbal context in the cartoon. To test this hypothesis, we compared the patterns of connectivity and time differences in MEG parameters in the various brain regions within and between the hemisphere regarding tasks for MGJs and MBJs.

## Materials and Methods

### Participants

Fifteen healthy male volunteers from Kanazawa University were recruited for this study. All provided written informed consent to participate in the experiment, which was approved by the Ethical Committee of Kanazawa University, and this study was performed in accordance with the Declaration of Helsinki. Because four participants were excluded due to head motion artifacts in their MEG data (*n* = 2) and low behavioral performance (less than 70% accuracy) (*n* = 2), data from eleven subjects were fully analyzed. All 11 participants (mean = 21.7 years old, SD = 1.32) were right-handed, native Japanese speakers with normal or corrected-to-normal vision and were not taking antidepressant medication. None of the participants had prior or current neurological or psychiatric disorders (e.g., traumatic brain injury with loss of consciousness, epilepsy, neurological impairment, or degenerative neurological illness), as ascertained by a detailed anamnesis. The full IQ score among the 11 subjects was within a normal range (mean = 108.9, SD = 4.63) using the Japanese version of the National Adult Reading Test (Matsuoka et al., 2006).

### Task

**FIGURE 1:**
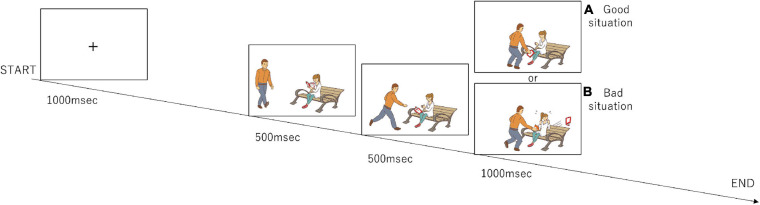
Design of the moral judgment task. The participants were presented three-frame video clips about morally positive, negative, and neutral contexts and were asked to judge morality as soon as possible after the presentation of the third picture. The numbers of presentations for the positive, negative, and neutral stories are 96, 96, and 48, respectively. **(A)** The third frame that represents a morally good situation. **(B)** The third frame that represents a morally bad situation.

The participants completed a set of tasks (Figure 1) that were modified from the previous study by Decety and Cacioppo (2012). During the task, the participants watched a series of three-frame video clips that were presented centrally on a monitor screen. Before a story began, a fixation cross appeared for 1,000 msec. Following the fixation cross, the first frame and the second frame from the video clip, which were each 500 msec long, were displayed to establish the scene; the third frame (Phase 3) was 1,000 msec long and displayed a scene requiring a moral judgment. After Phase 3 disappeared, the question “Do you think this was good or bad?” in Japanese was displayed for 1,000 msec. The participants were asked to judge by pressing a button with a right thumb if a behavior of a person in pictures was considered to be morally good or pressing a button with a left thumb if morally bad during a period of 1,000 msec. If they judged it as a morally neutral behavior, they did not push any button. The same number of good and bad situations were presented in Phase 3 (96 situations each), and the number of neutral situations was 48. They were presented in a random order.

### MEG

We used a MEG system that had a 160-channel whole-head gradiometer equipped with coaxial-type gradiometers (MEG vision PQA160C; RICOH Company, Ltd., Kanazawa, Japan) set in a shielded room (Daido Steel, Nagoya, Japan). MEG data were recorded by using MEG-160 software (RICOH Company) with a sampling rate of 1,000 Hz. A T1-weighted brain MRI image was acquired for every subject with spherical liquid markers placed at the five MEG fiducial points using a Signa Excite HD 1.5T system (GE Yokogawa Medical Systems Ltd., Milwaukee, WI, United States), which enabled us to superimpose the MEG coordinate system on the MRI data. T1-weighted images consisted of 166 sequential 1.2 mm-thick slices with a resolution of 512 × 512 points within a field of view of 261 mm × 261 mm. The cortex surface was reconstructed using FreeSurfer software (version 5.3^1^).

## Analysis and Statistics

### Behavioral Data

Since we considered that an individual’s morality would alter response times, a paired *t*-test was used to compare mean response times for moral judgments. A significance threshold of *p* < 0.05 was used for all comparisons.

### MEG Data

Magnetoencephalography data preprocessing was performed using MEG-160 software (RICOH Company, Ltd., Kanazawa, Japan). The recorded MEG data were resampled to 500 Hz and filtered by a 0.1–115 Hz bandpass filter and notch (60 Hz) filter. After preprocessing, MEG data were processed using Brainstorm (Tadel et al., 2011) on MATLAB R2017b (The Math works, Natick, MA, United States). MEG data were extracted from 200 ms before and 1,000 ms after the beginning of the third frame (Phase 3) (total 1,200 ms) for every trial. The periods with false responses were rejected from the analysis. The frequency bands were divided into delta (0.2–3 Hz), theta (4–7 Hz), alpha (8–13 Hz), beta (14–31 Hz), and gamma (32–100 Hz) bands for this time-frequency analysis. Similarly, in the time-frequency analysis, based on the previous study that harmful information was processed through the posterior STS (62–140 ms), amygdala (122–180 ms) and ventro-mPFC (182–304 ms) (Decety and Cacioppo, 2012), we analyzed changes in functional connectivity over time in our data during the same time periods examined in their study. In addition, we analyzed functional connectivity in our data from −200 ms to 1,000 ms in 100-ms epochs in all channels. The software Brainstorm (Tadel et al., 2011) on MATLAB R2017b (The Math works, Natick, MA, United States) was used for this connectivity analysis. Statistical significance of brain activity for 1ms as minimum duration was considered as significance level alpha (*p*-value) was less than 0.05, and the Bonferroni correction was used for post hoc analyses.

## Results

### Behavior

While the mean response time for MBJs (2.24 s ± 0.37) tended to be longer than the mean time for MGJs (2.19 s ± 0.39), there was no significant difference between them (*t* = 0.297, df = 20, *p* = 0.770, *d* = 0.13). Likewise, there were no differences between the mean correct response rates among these three judgments [*F*_(2,30)_ = 1.614, *p* = 0.216, η^2^ = 0.10] [MGJ = 92.6% (SD = 7.6), MBJ = 91.1% (SD = 9.0), MNJ = 97.0% (SD = 5.8)].

### Brain Activity

#### Brain Activation

Comparisons of the MGJ and MBJ conditions from −200 to 1,000 ms with a minimum duration of 1 ms showed 12 channels with significant differences at certain times (Table 1). All channels except LT31 and LT41 showed significantly higher activations during MGJs than during MBJs from 564 to 810 ms. The observed differences during this period indicated that these functional brain areas on the right hemisphere were activated by MGJs and not MBJs. Channels LT31 and LT41 showed significantly higher activation during MBJ than during MGJ from 664 to 734 ms, indicating that these functional brain areas in the left temporal area were activated by MBJs rather than MGJs during this period.

**TABLE 1:** Significant activation across channels.

**Time (ms)**	**Area**	**CH**	**Good vs Bad**
564	RostralMiddleFrontal	RF33	Good
598	CaudalMiddleFrontal	RF34	Good
600	RosttralMiddleFrontal	RF33	Good
624	Parstriangularis	RT11	Good
634	Parstriangularis	RT11	Good
636	SuperiorFrontal	RC21	Good
	RosttralMiddleFrontal	RF33	
638	SuperiorFrontal	RF41 and RF42	Good
	SuperiorParietal	RC35	
640	PreCentral	RC34	Good
	SuperiorParietal	RC35	
658	SuperiorFrontal	RC21	Good
664	SuperiorTemporal	LT31	Bad
668	AnteriorTemporal	LT41	Bad
672	SuperiorFrontal	RC21	Good
690	SuperiorFrontal	RF21	Good
710–712	SuperiorFrontal	RF41	Good
724	SuperiorFrontal	RF21, RF41, LF31, and LF52	Good
734	AnteriorTemporal	LT41	Bad
738	SuperiorFrontal	RF41	Good
810	PostCentral	LC25	Good

*Comparisons of the power of the magnetic fields in MGJ and MBJ conditions from −200 to 1000 ms with a minimum duration of 1 ms (*p* < 0.05, Bonferroni-corrected multiple comparisons). The names of the brain areas were based on the Desikan-Killiany atlas in the Brainstorm software.*

#### Time-Frequency Analysis

**FIGURE 2:**
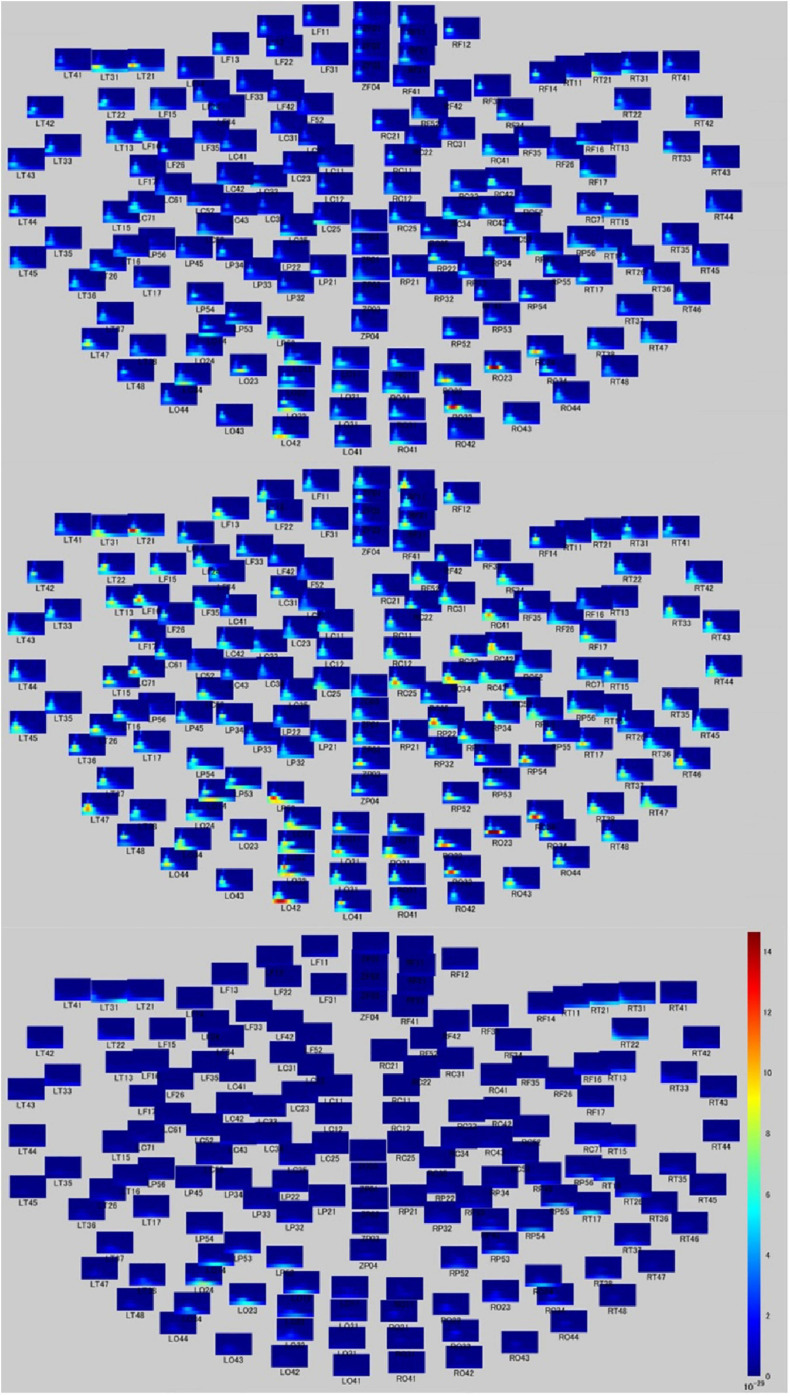
Time-frequency relationship map during moral judgment. Time-frequency figures on whole head during MGJ (upper), MBJ (middle), and MNJ (lower) conditions. The *X*-axis of each small panel indicates the time from 200 ms before to 1,000 ms after a phase three picture presentation, and the *Y*-axis indicates the Hz from 0 to 120. The color bar denotes the power (signal units 2/Hz × 10–29) from 0 to 15.

Time-frequency analyses showed differential band activity in each moral judgment situation. In the MGJ condition, channels LT21, LT31, LT47, RO23, RO24, and RO32 (covering the left temporal area and the right occipital area) showed a higher rate of theta band activity, and channels RT21, LO12, LO32, and LO42 (covering the right temporal area and the left occipital area) showed a higher rate of delta band activity after presentation of Phase three. In the MBJ condition, channels LO32 and LO42 (covering the left occipital area) showed a higher rate of delta band activity, and channels RO22, RO23, RO24, RO32, LP52, LT47, LC71, LF16, and LT21 showed a higher rate of theta band activity. During MNJ conditions, there was a low rate of delta band activity in channels LO14, RT17, and RO24 (Figure 2).

#### Connectivity and Its Temporal Changes

**FIGURE 3:**
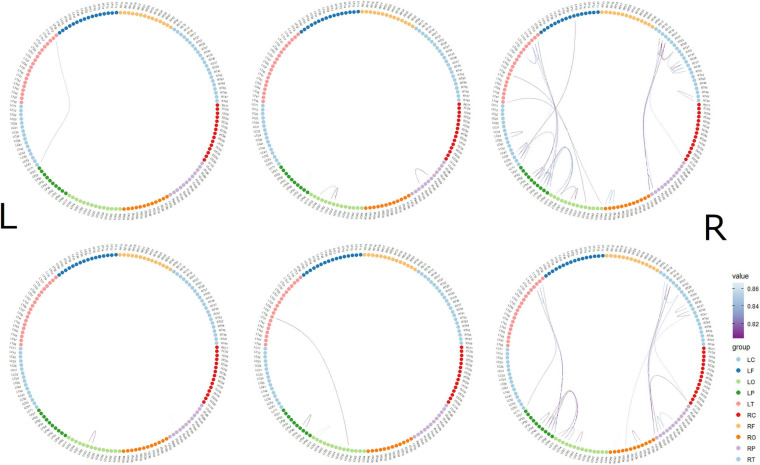
Time-dependent changes in functional connections. The top row shows morally bad judgment conditions and bottom row shows morally good judgment conditions. The left column shows 62–140 ms, middle column shows 122–180 ms, and right column shows 182–304 ms. L indicates left hemisphere and R indicates right hemisphere. MEG channels are placed on RF (right frontal area), RT (right temporal area), RC (right central area), RP (right parietal area), RO (right occipital area), LO (left occipital area), LP (left parietal area), LC (left central area), LT (left temporal area), and LF (left frontal area). The value denotes the correlation coefficient r.

The top 10 short-distance channel pairs, along with their correlations and distances, that represented the highest correlation coefficients (*r*-values) that were found during MGJ and MBJ conditions (0 –1000 ms) are shown in Table 2; all channel pairs except one were found in the left hemisphere during MBJ conditions, and the number of pairs involving the LT21 channel was higher during MBJ than MGJ conditions. This indicated that fast information processing likely occurred in the neighboring brain regions in the left hemisphere during MBJs. In Table 3, the top 10 channel pairs during MGJ and MBJ conditions (0–1000 ms) are shown and had low correlations (*r* < 0.607). These low correlation coefficients indicated that remote or interhemispheric neural parallel processing was not significant during moral judgment irrespective of MGJ or MBJ conditions. Tables 4, 5 and Figure 3 show time-dependent changes in functional connections during moral judgments. The top row is from MBJ conditions (bad), and the bottom row is from MGJ conditions (good); The left column shows 62–140 ms, the middle column shows 122–180 ms, and the right column shows 182–340 ms; orange lines represent functional connections (0.8 < r) and their channel pairs. These time-frequency results indicated that the number of functional connections increased with time and that the connections within the left hemisphere were primarily observed in MBJ conditions.

**TABLE 2:** Connectivity with high intensity among the channel pairs.

**Judgment**	**correlation (*r*)**	**CH pair**		**Distance (mm)**
Good	0.933	LT21	LT31	26
Good	0.921	RT21	RT22	23
Good	0.906	RT22	RT31	33
Good	0.902	LT22	LT31	36
Good	0.902	RT21	RT31	26
Good	0.894	RT11	RT31	50
Good	0.894	LT21	LT22	26
Good	0.89	LT21	LT42	58
Good	0.89	LT31	LT42	35
Good	0.886	LT21	LT33	57
Good	0.886	LT31	LT33	47
Bad	0.87	LT21	LT22	26
Bad	0.833	RT31	RT41	27
Bad	0.83	LT21	LF15	49
Bad	0.825	LT21	LT31	26
Bad	0.816	LT15	LT22	42
Bad	0.802	LT21	LF14	44
Bad	0.793	LF14	LF15	25
Bad	0.793	LF15	LF16	23
Bad	0.79	LT21	LT13	52
Bad	0.788	LT21	LT33	57

*Channel pairs and their distances that showed the top 10 highest correlations (*r*-values) for each judgment.*

**TABLE 3:** Connectivity with long distance among the channel pairs.

**Judgment**	**Correlation (*r*)**	**CH pair**		**Distance (mm)**
Good	0.552	LF16	RF31	147
Good	0.554	LT21	RF31	143
Good	0.678	LT31	LT47	142
Good	0.556	RF34	RT41	141
Good	0.603	LF16	LT47	138
Good	0.579	LT41	LT47	138
Good	0.583	LF13	LT44	136
Good	0.623	LT22	LT47	132
Good	0.556	LF15	RF31	132
Good	0.552	LT13	LT38	130
Bad	0.619	RF21	LF26	149
Bad	0.662	RF11	LF16	147
Bad	0.63	RF52	LF15	146
Bad	0.607	RF12	LF15	146
Bad	0.59	ZF02	LF17	146
Bad	0.699	RF21	RT41	144
Bad	0.599	LC71	LF31	144
Bad	0.676	ZF01	RT41	143
Bad	0.625	RC21	LF17	143
Bad	0.607	RC22	LF31	143

*The top 10 long distance channel pairs during MGJ and MBJ conditions with correlation coefficients (*r*-values).*

**TABLE 4:** Time-dependent changes in significant functional connections during MGJs.

	**62–140 ms**		**122–180 ms**		**182–340 ms**	
1	LO14	LO24	LC61	LC52	LO14	LP54
2	LO23	LO24	LC71	LT15	LO23	LO24
3	LC71	LT15	LO11	LO12	LO24	LO23
4	RP53	RP54	LO34	LT38	LP52	LP53
5	RP56	RT16	LP52	LO12	LP53	LO14
6			LP52	LP53	LP53	LP54
7			LP53	LO14	LT16	LP56
8			LP53	LP54	LT16	LT17
9			LT16	LT26	RO24	RO23
10			RT26	RT16	RP43	RP53
11			RT26	RT36	RP53	RP54
12					RP55	RT17
13					RP56	RT16
14					RT15	RC71
15					RT17	RT16
16					RT26	RT16
17					RT36	RT35
18					RT37	RT38

*Channel pairs showing correlation coefficient *r*-values over 0.8 in each time domain during MGJs. Time windows are in reference to Decety and Cacioppo (2012).*

**TABLE 5:** Time-dependent changes in significant functional connections during MBJs.

	**62–140 ms**		**122–180 ms**		**182–340 ms**	
1	LT15	LC71	LO14	LO24	LC34	LC35
2			LO23	LO24	LC52	LC53
3			LP53	LP54	LC53	LP34
4			RP43	RP53	LC61	LC71
5					LC61	LC52
6					LC71	LT15
7					LO14	LO24
8					LO14	LP54
9					LO23	LO24
10					LO34	LT38
11					LO44	LT48
12					LP33	LP34
13					LP45	LP34
14					LP45	LP56
15					LP45	LT15
16					LP52	LO12
17					LP52	LO14
18					LP52	LP53
19					LP53	LO14
20					LP53	LP54
21					LP54	LT17
22					LP56	LC71
23					LT16	LC71
24					LT16	LP56
25					LT16	LT26
26					LT17	LT26
27					RO42	RO43
28					RO43	RO44
29					RP55	RT17
30					RP56	RP55
31					RP56	RT16
32					RP56	RT17
33					RT37	RT38
34					RT37	RT36
35					RT15	RC71
36					RT15	RT17
37					RT17	RT16
38					RT26	RT36
39					RT36	RT35
40					RT47	RT48

*Channel pairs showing correlation coefficient *r*-values over 0.8 in each time domain during MBJs. Time windows are in reference to Decety and Cacioppo (2012).*

## Discussion

In this study, we showed brain activations in different regions and that there was different intensities [correlation coefficients (*r*-values)] of time-dependent neural connectivity between the MGJ and MBJ conditions. While the brain activation patterns were generally similar between them, many regions in the right hemisphere were involved in MGJs, especially highlighting regions related to emotion and “theory of mind” processes. In contrast, the left hemisphere was more implicated in MBJs, where the neural connections between neighboring regions in an earlier decision period was likely characteristic of the MBJ process.

A hemispheric difference in cognitive processes between MGJs and MBJs is interesting. While it was reported that left-lateralized brain activity was predominant during MBJs (Cope et al., 2010), the number of activated channels was limited, and local areas on the left hemisphere (LT31 and LT41) were found to be activated in MBJ conditions in our study (see Table 1). The regions associated with these channels (anterior temporal lobe) are considered to subserve simple moral judgments (Moll et al., 2001, 2002a) and social cognition by providing abstract conceptual knowledge of social behaviors (Zahn et al., 2007). Therefore, the MBJ process may be mediated in more local, temporal regions in the left hemisphere, which are engaged in simple decisions that have social implications. In contrast to the MBJ conditions, in the MGJ conditions, many channels covering the frontal, central and temporal areas on the right hemisphere were activated, as shown in Table 1. The right frontal cortex is important in the integration of emotion into decision-making and planning (Partiot et al., 1995; Reiman et al., 1997) and in the regulation of “theory of mind” (Castelli et al., 2000; Frith, 2001). It was also reported that the right hemisphere is important in intent-based moral judgments from an experiment with a split-brain patient (Steckler et al., 2017). Thus, MGJs may incorporate many right hemisphere regions that relate to moral judgment under the influence of empathy, emotional planning, and intention.

**FIGURE 4:**
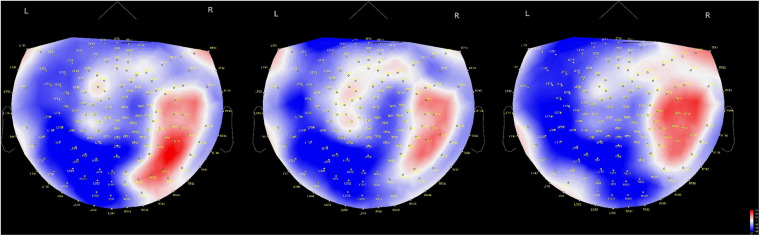
Time-dependent changes in activated brain areas on 2D cap images. Time windows: left shows 62–140 ms, middle shows 122–180 ms and right shows 182–304 ms. L, left hemisphere; R, right hemisphere. The color bar denotes amplitude (fT) from –60 (blue) to 60 (red).

Regarding the time course of processing of moral judgments when viewing the scenes, brain activation is likely to proceed from the occipital to frontal cortex during moral judgment, as shown in Figure 4, which is consistent with a previous report in which a visually presented unpleasant story similar to our MBJ task sequentially activated brain regions through the posterosuperior temporal sulcus to the amygdala to the ventromedial prefrontal cortex in an EEG study (Decety and Cacioppo, 2012). Theta waves reflects states of contemplation, and delta waves are an indicator of attention to internal processing while performing mental tasks (Harmony et al., 1996). Channels with significant theta activation commonly found between the MGJ and MBJ conditions were on the left temporal pole and right occipital area in our study. Because these activated theta bands appeared around the start of the presentation of Phase 3, these channels might be involved in mentalization in the same process. Mentalization refers to the ability to read the mental states of other agents and engages many neural processes (Frith and Frith, 2006). In the present study, the participants were asked to show their mentalizing ability for reading the minds of protagonists in the pictures on the basis of morality irrespective of MGJ or MBJ conditions. In fMRI studies, tasks that required mental state reasoning reportedly activated brain regions, including the temporoparietal junction, superior temporal sulcus, anterior temporal and dorsomedial prefrontal cortices (Mar, 2011; Schurz et al., 2014; Molenberghs et al., 2016). Thus, again, the channels activated here can be considered regions important for a mentalizing network. Some channels on the left hemisphere from the occipital to parietal area followed by the channels on the right central area exhibited theta activation only during MGJ conditions in a time-dependent manner. Therefore, in MGJ conditions, there was a fluctuation of activation across the hemisphere with time. In the case of delta waves, occipital regions in both the MBJ and MGJ were identified as having a higher level of delta power, as shown in Figure 2. When participants were asked to judge a morality of a presented story, they need to recall moral knowledges that they already got, apply them to a faced situation and judge a morality of it. Information flow between the left inferior frontal gyrus (IFG) and the left STS gyrus suggested an existence of feedback loop (Kitaura et al., 2017). Because strong theta activation during MBJ and weak delta activation during MGJ were seen in both the left anterior temporal lobe and the left parietal area, there is a possibility that our delta activation reflects participants’ feedback loop for moral knowledge recall for moral judgment. These suggested that viewing pictures for morality in our situation might recall moral knowledge in the feedback loop and enhance internal processing of attention to morality in the occipital cortex irrespective of the valence of the condition.

In the present study, there was a difference in functional connectivity between the MBJ and MGJ conditions. Functional connectivity between brain regions is defined as the temporal correlation between spatially remote neurophysiological events (Friston et al., 1993). The distances of the top 10 high intensity [i.e., high correlation coefficients (*r*-values)] channel pairs during both MGJ and MBJ conditions ranged from 23 mm to 58 mm, indicating that the channel pairs were located close to each other. Since MEG detects signals mainly from neurons under the detectors, the channels with similar waves imply that brain areas with these channels function in the same fashion. Because LT21 and its paired channels on the left temporal cortex showed high intensity [i.e., high correlation coefficients (*r*-values) during both MGJ and MBJ conditions, this area is important as a source of neural connection in moral judgment. In contrast, the temporal and frontal cortices with short mutual connections showed strong connectivity during MBJs but not MGJs. The time-frequency analysis showed that LT21 and LF16 commonly showed theta activation in MBJ conditions. Channels LF14, LF15, and LF16 covering the left orbitofrontal/ventromedial frontal cortex and activated during MBJs are considered to relate to simple moral judgments (Moll et al., 2001, 2002a), reward/punishment (O’Doherty et al., 2001) and emotion-related information processing (Fink et al., 1996; Lane et al., 1997; Reiman et al., 1997; Blair et al., 1999). Therefore, these findings suggested that MBJs are accompanied by stronger functional connectivity within the limited temporal cortex under the influence of other factors (i.e., reward/punishment calculation) than those accompanying MGJs.

It is well acknowledged that an inappropriate response to a morally negative situation degrades oneself as being morally defective, which would make processing during MBJs more crucial and faster than that during MGJs in daily life. It was reported that neural activation in the left temporal parietal junction was greater when a protagonist told lies for antisocial than for prosocial purposes (Harada et al., 2009). Consistent with this judgment of lying, MBJs similarly activated the left temporal cortex within a shorter duration and with tighter neuronal connections in the neighboring brain regions. Unlike the neural activation in the MGJ conditions, which showed broader involvement of the brain region to make a judgment, the local region (temporal cortex) activated during MBJs might be advantageous in responding to an encountered situation within a shorter time. In a previous EEG study with predominantly female participants, a picture presentation with morally negative information generated a temporal change in activation from the posterosuperior temporal sulcus region to the ventromedial prefrontal cortex via the amygdala in the right hemisphere (Decety and Cacioppo, 2012). This time-dependent change in activation from posterior to anterior brain regions was duplicated in our study (Figure 4). However, our MEG was not able to evaluate amygdalar activation during MBJ conditions. Nevertheless, amygdala involvement in the left hemisphere in the rapid MBJ process might be likely because the amygdala is implicated in emotion-involved information processing. Although our tasks are same as Decety and Cacioppo (2012) that a participant observes a presented moral story and judges its morality from the viewpoint of a third parity who looks a moral situation, their EEG study reported that visually presented negative moral information is processed from the pSTS to the vmPFC via the amygdala (Decety and Cacioppo, 2012). Moreover, previous moral studies suggested that emotion plays a role during moral judgment (Moll et al., 2002b; Haidt, 2003; Tangney et al., 2007; Harenski et al., 2009; Gray and Wegner, 2011; Krettenauer and Johnston, 2011). When considering the neural activity related to emotional processing, previous studies have shown that relative left-hemispheric activation for positive emotions and relative right-hemispheric activation of negative emotion in frontal brain areas (Ahern and Schwartz, 1985) and that overall brain activity was lateralized towards the left hemisphere for positive pictures and towards the right hemisphere for negative pictures when the experience of valence was equated for arousal (Canli et al., 1998). Orbito-frontal cortex (OFC) plays an important role not only in reward coding of environmental sensory cues but also in behavioral planning and decision-making (Tanji and Hoshi, 2001; Wallis, 2007; Young and Shapiro, 2011). Because RF11 activation on the right OFC during MBJ was higher than that during MGJ, this difference would reflect the OFC’s role in value-based decision-making. Moreover, CHs that located on the left hemisphere from occipital area to parietal area and CHs that located on the right central area showed significant theta activation on only during MGJ shown in Figure 2. These suggested that this different activation pattern between MGJ and MBJ reflects moral judgment based on reward- and/or emotion-related information processing.

There are several limitations in the present study. First, the participants were all men. Indeed, there are some sex differences in morality (Gilligan, 1977). Since it has been reported that women are more self-focused and men are more world-focused in thinking (Moriguchi et al., 2014), we did not recruit women here because the purpose of the present study was to examine differences in temporal and regional patterns of neuronal processing estimated with MEG under the emotionally inflicted condition such as guilt in order to show the MEG availability on these two aspects simultaneously, and also avoid confounding factors originating from sex-related differences in emotion processing. Second, the number of participants was small. Thus, we used a conservative threshold for significance in an MEG study to reduce the false-negative rate in this study. Actually, sample numbers around 10 are acceptable in recent studies with MEG (Lankinen et al., 2018; Paraskevopoulos et al., 2018; Youssofzadeh and Babajani-Feremi, 2019; Bonaiuto et al., 2020; Weber et al., 2021). Third, there were no elderly participants. Moral judgment depends considerably on personal experiences occurring in social interactions. Indeed, since moral judgment can be defined as the evaluation of actions with respect to norms and values established in a society, moral judgment likely embraces an aspect of judgment of a behavior whether it is socially acceptable or unacceptable. Therefore, the present findings cannot be extended to other generations, even in the male population. Thus, a future study with a larger sample size and a mixture of people (men and women) across a broader range of generations is needed to explore more about differences in moral judgment processing with age and sex.

## Conclusion

The present activation analysis and connectivity analysis showed that MBJs activated localized left temporal regions with stronger connectivity between brain areas related to emotion and reward/punishment than MGJs. In the MGJ conditions, the broader range of brain regions subserving emotion and “theory of mind” were activated predominantly in the right hemisphere. Furthermore, time-frequency analysis showed that the left hemisphere was more crucial for MBJs than for MGJs in early moral information processing and that intrahemispheric information processing was more implicated than interhemispheric information processing up to 304 ms during MBJs. These results suggested that MBJs require rapid information processing working in a specialized brain region (the temporal cortex in this case) to avoid disgraceful circumstances without any delay.

## Data Availability Statement

The datasets generated during and/or analyzed during the current study are not publicly available due to the absence of agreement of the participants, but they are available from the corresponding authors on reasonable request.

## Ethics Statement

The studies involving human participants were reviewed and approved by the Ethical Committee of Kanazawa University. The patients/participants provided their written informed consent to participate in this study.

## Author Contributions

HH designed the study. HH, TI, CH, DS, and SK conducted the data curation. HH and TI conducted the data analyses. MK supervised the research. YO revised the manuscript. All authors contributed to the article and approved the submitted version.

## Conflict of Interest

The authors declare that the research was conducted in the absence of any commercial or financial relationships that could be construed as a potential conflict of interest.

## Footnotes

1
http://surfer.nmr.mgh.harvard.edu/
