# Corrigendum: Regional and Temporal Differences in Brain Activity With Morally Good or Bad Judgments in Men: A Magnetoencephalography Study

**DOI:** 10.3389/fnins.2021.753147

**Published:** 2021-10-21

**Authors:** Hirotoshi Hiraishi, Takashi Ikeda, Daisuke N. Saito, Chiaki Hasegawa, Sachiko Kitagawa, Tetsuya Takahashi, Mitsuru Kikuchi, Yasuomi Ouchi

**Affiliations:** ^1^Department of Biofunctional Imaging, Preeminent Medical Photonics Education and Research Center, Hamamatsu University School of Medicine, Hamamatsu, Japan; ^2^Research Center for Child Mental Development, Kanazawa University, Kanazawa, Japan; ^3^United Graduate School of Child Development, Osaka University, Kanazawa University, Hamamatsu University School of Medicine, Chiba University and University of Fukui, Osaka, Japan; ^4^Department of Psychology, Yasuda Women's University, Hiroshima, Japan; ^5^Department of Psychiatry and Behavioral Science, Kanazawa University, Kanazawa, Japan

**Keywords:** moral judgment, MEG, brain activity, connectivity, morally good judgment, morally bad judgment

In the original article, there was a mismatch between the figures and their legends as published. The captions for Figures 1 and 2 were switched, and the captions for Figures 3 and 4 had also switched in the published article. As a result, all the figures and their figure legends were mismatched. To resolve this, the image currently used for Figure 2 should instead be Figure 1, and the image currently used for Figure 1 should instead be Figure 2. Similarly, for Figures 3 and 4, the images should be swapped, so that the image currently labeled as Figure 3 becomes Figure 4, and the image labeled as Figure 4 becomes Figure 3. The captions are then correct as they are. The correct figures appear below.

**Figure 1:**
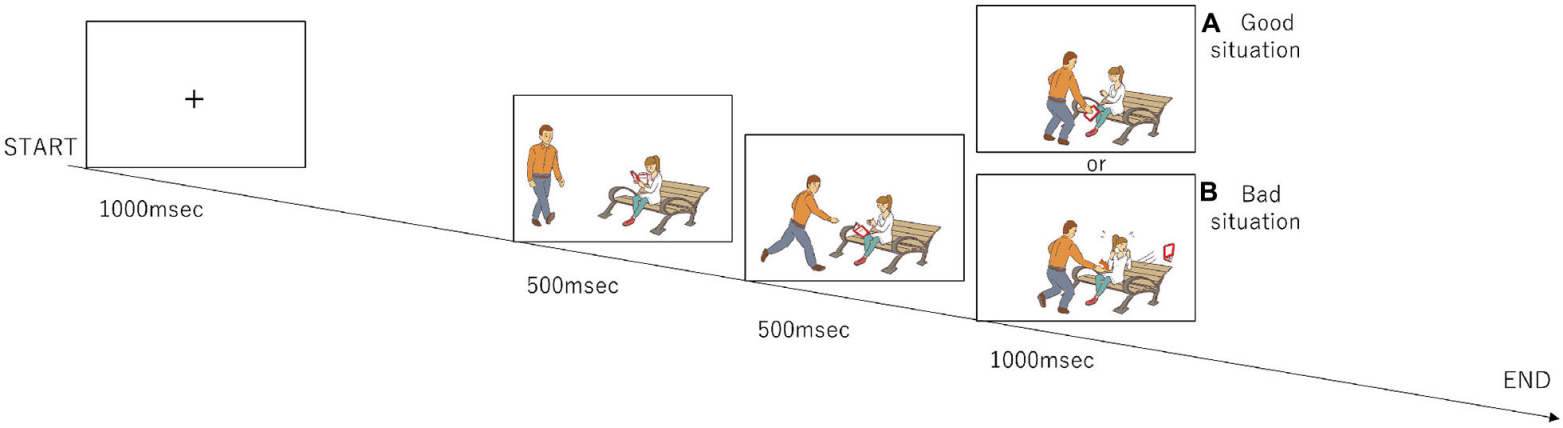
Design of the moral judgment task. The participants were presented three-frame video clips about morally positive, negative, and neutral contexts and were asked to judge morality as soon as possible after the presentation of the third picture. The numbers of presentations for the positive, negative, and neutral stories are 96, 96, and 48, respectively.

**Figure 2:**
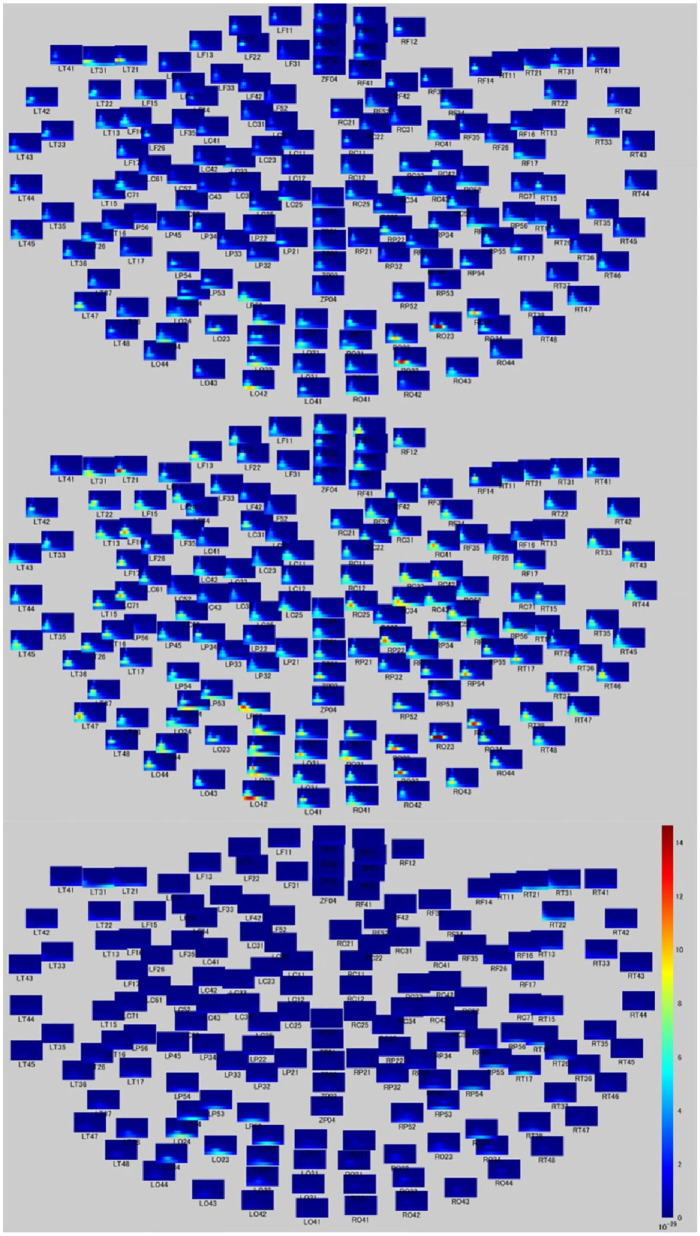
Time-frequency relationship map during moral judgment. Time-frequency figures on whole head during MGJ (upper), MBJ (middle), and MNJ (lower) conditions. The *X*-axis of each small panel indicates the time from 200 ms before to 1,000 ms after a phase three picture presentation, and the *Y*-axis indicates the Hz from 0 to 120. The color bar denotes the power (signal units 2/Hz × 10–29) from 0 to 15. **(A)** The third frame that represents a morally good situation. **(B)** The third frame that represents a morally bad situation.

**Figure 3:**
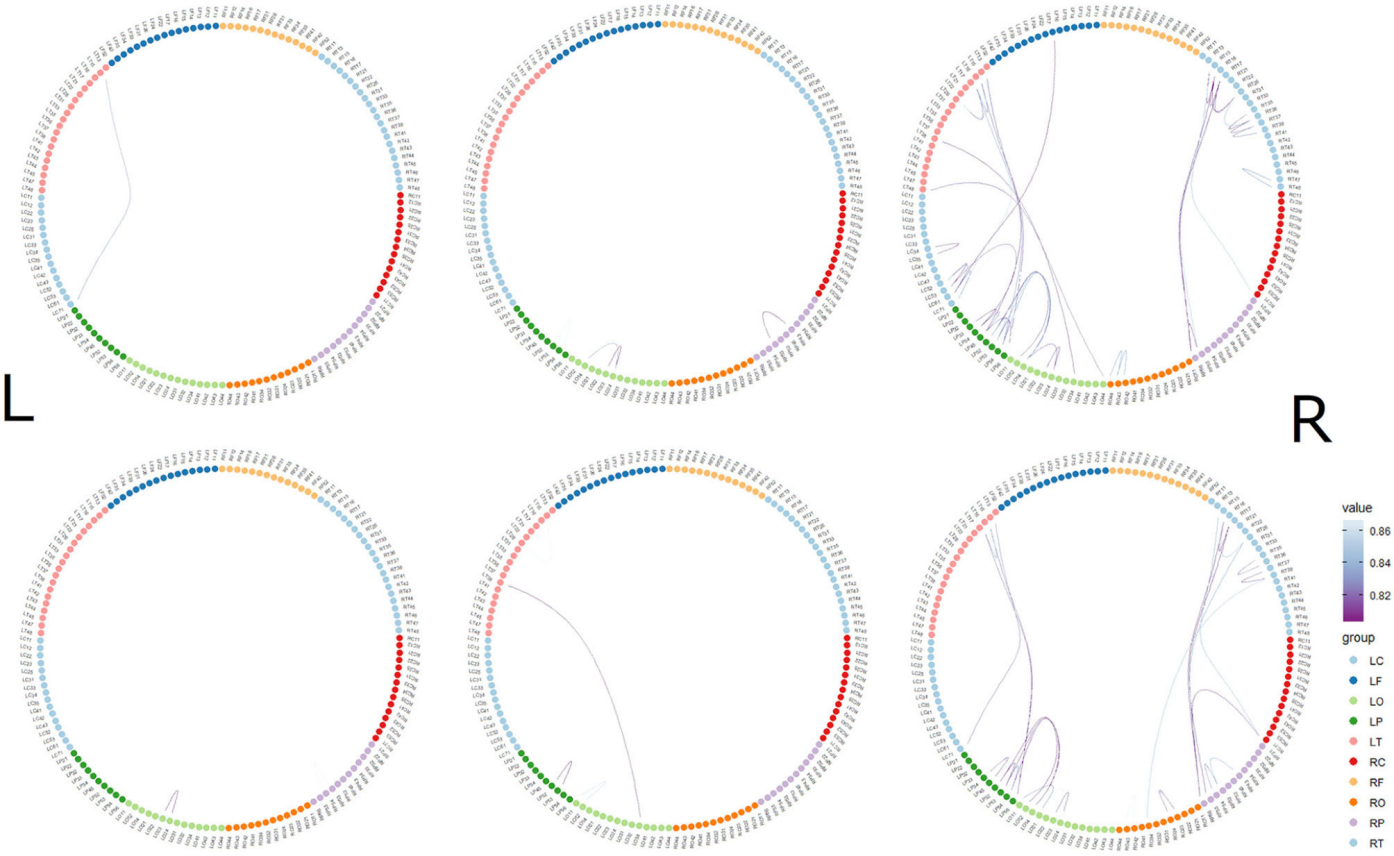
Time-dependent changes in functional connections. The top row shows morally bad judgment conditions and bottom row shows morally good judgment conditions. The left column shows 62–140 ms, middle column shows 122–180 ms, and right column shows 182–304 ms. L indicates left hemisphere and R indicates right hemisphere. MEG channels are placed on RF (right frontal area), RT (right temporal area), RC (right central area), RP (right parietal area), RO (right occipital area), LO (left occipital area), LP (left parietal area), LC (left central area), LT (left temporal area), and LF (left frontal area). The value denotes the correlation coefficient r.

**Figure 4:**
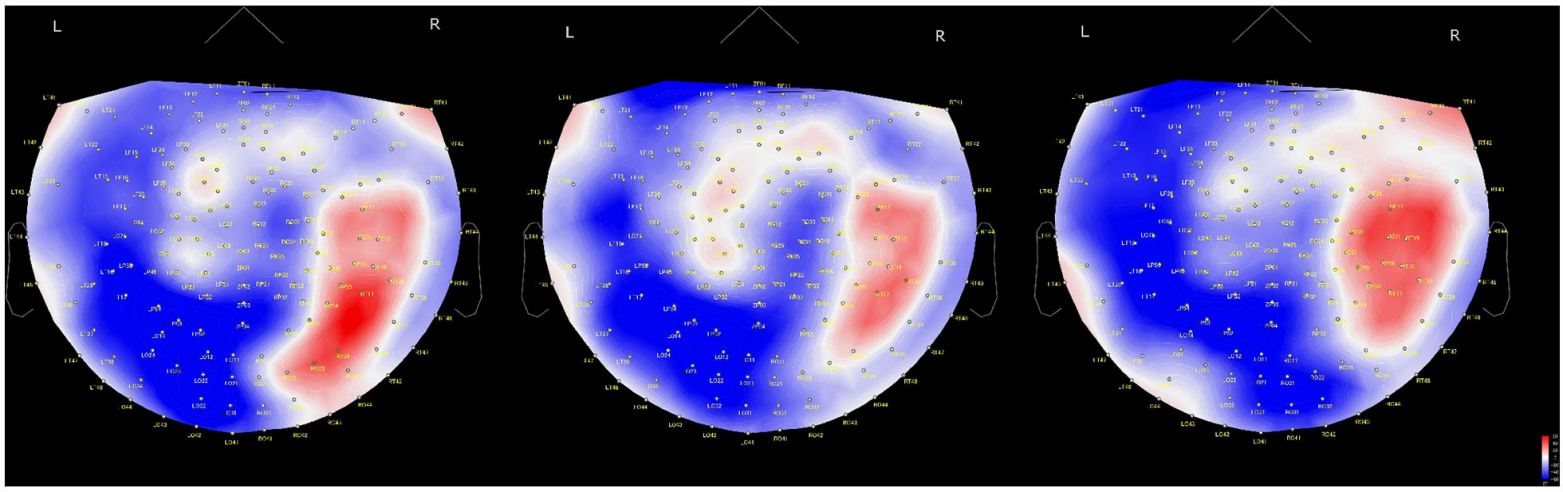
Time-dependent changes in activated brain areas on 2D cap images. Time windows: left shows 62–140 ms, middle shows 122–180 ms and right shows 182–304 ms. L, left hemisphere; R, right hemisphere. The color bar denotes amplitude (fT) from –60 (blue) to 60 (red).

Additionally, in the published article, there was an error in affiliation 3 as published. Instead of “United Graduate School of Child Development, Osaka University, Kanazawa University, Hamamatsu University School of Medicine, Chiba University and University of Fukui, Fukui, Japan”, it should be “United Graduate School of Child Development, Osaka University, Kanazawa University, Hamamatsu University School of Medicine, Chiba University and University of Fukui, Osaka, Japan”.

Lastly, in the original article, there was a duplication of a description provided in the Materials and Methods section. The following sentence appearing at the end of the this section has been removed:

“The same number of good and bad situations were presented in Phase 3 (96 situations each), and the number of neutral situations was 48. They were presented in a random order.” The corrected paragraph is shown below.

The participants completed a set of tasks (Figure 1) that were modified from the previous study by Decety and Cacioppo (2012). During the task, the participants watched a series of three-frame video clips that were presented centrally on a monitor screen. Before a story began, a fixation cross appeared for 1,000 msec. Following the fixation cross, the first frame and the second frame from the video clip, which were each 500 msec long, were displayed to establish the scene; the third frame (Phase 3) was 1,000 msec long and displayed a scene requiring a moral judgment. After Phase 3 disappeared, the question “Do you think this was good or bad?” in Japanese was displayed for 1,000 msec. The participants were asked to judge by pressing a button with a right thumb if a behavior of a person in pictures was considered to be morally good or pressing a button with a left thumb if morally bad during a period of 1,000 msec. If they judged it as a morally neutral behavior, they did not push any button. The same number of good and bad situations were presented in Phase 3 (96 situations each), and the number of neutral situations was 48. They were presented in a random order.

The authors apologize for these errors and state that they do not change the scientific conclusions of the article in any way. The original article has been updated.

## Publisher's Note

All claims expressed in this article are solely those of the authors and do not necessarily represent those of their affiliated organizations, or those of the publisher, the editors and the reviewers. Any product that may be evaluated in this article, or claim that may be made by its manufacturer, is not guaranteed or endorsed by the publisher.
